# Effects of an intergenerational Kindermusik class on stress and affect in older adults with dementia and their caregivers: a pilot study using ecological momentary assessment

**DOI:** 10.3389/fragi.2025.1448293

**Published:** 2025-06-17

**Authors:** Amber Thro, Phyllis Babcock, T. Lee Covington, Kelly Green, Carol Penney, Jason Fanning, Christina E. Hugenschmidt

**Affiliations:** ^1^ Gerontology and Geriatric Medicine, Wake Forest School of Medicine, Winston-Salem, NC, United States; ^2^ Atrium Health Wake Forest Baptist, Winston-Salem, NC, United States; ^3^ Senior Services, Inc., Winston-Salem, NC, United States; ^4^ Kindermusik International, Greensboro, NC, United States; ^5^ Department of Health and Exercise, Wake Forest University, Winston-Salem, NC, United States

**Keywords:** dementia, caregiver, music, ecological momentary assessment, Kindermusik

## Abstract

**Introduction:**

The objectives of this pilot study were to determine the feasibility of collecting ecological momentary assessments from caregivers of older adult persons with dementia as they attended a weekly, intergenerational Kindermusik class, and to examine relationships between Kindermusik participation and momentary stress and affect for the caregiver and person with dementia.

**Methods:**

Over a 12-week period, 14 persons with dementia attended a weekly intergenerational Kindermusik class. Caregivers completed up to six daily ecological momentary assessments on affective valence and stress for themselves and the person with dementia during Weeks 1 and 12. Salivary cortisol data were collected from the persons with dementia during Weeks 1 and 12.

**Results:**

Overall, ecological momentary assessment response rates were low (37.7% completion). Caregiver ecological momentary assessment ratings of their personal stress and affect were significantly and positively associated with their perceptions of affect and stress in the person with dementia. The caregiver’s affective state tended to improve on days when the person with dementia attended Kindermusik, especially when they perceived the person with dementia as having more positive affect.

**Discussion:**

Additional research is required to determine how to best support survey completion while minimizing caregiver burden, and to investigate the impact of more frequent involvement in an intergenerational music and movement program.

## 1 Introduction

Alzheimer’s disease and related dementias (ADRD) are neurodegenerative disorders that affect approximately 55 million people worldwide (World Health Organization, 2021). ADRD are characterized by cognitive impairments, such as memory loss, and behavioral and psychological symptoms of dementia (BPSD), including depression, anxiety, agitation, and aggression (Cerejeira et al., 2012). These symptoms significantly impact the quality of life of both persons with dementia (PWD) and their caregivers (Okura and Langa, 2011). A key focus in ADRD management is improving health-related quality of life, which encompasses physical, mental, and emotional wellbeing (Barbe et al., 2018). Emerging research identifies a number of promising non-pharmacological interventions for alleviating daily ADRD symptoms and enhancing quality of life in both PWD and their caregivers. For example, arts-based programs incorporating music and dance have shown evidence of improving cognition and reducing BPSD in PWD (Raglio et al., 2013; Särkämö et al., 2016). Music-based interventions, in particular, have been found to enhance memory recall, reduce the use of antipsychotic medications, and improve mood, especially when tailored to individual preferences (Inoue et al., 2024; Särkämö et al., 2016; Thomas et al., 2017). Similarly, intergenerational programs that bring together older adults and younger participants may enhance quality of life for PWD by fostering positive emotions while reducing stress and loneliness (Chung, 2009; Gerritzen et al., 2020).

One such example of an intergenerational program is Kindermusik Bridges, a community-delivered music and movement curriculum designed to foster engagement between preschool-aged children and older adults. Adapted from Kindermusik International, a widely used, research-based early childhood music program founded in the U.S. in 1978 and based on a German curriculum with influences from Orff, Kodaly, Vygotsky, and Montessori (Kindermusik, 2023; Winsler et al., 2011), Kindermusik Bridges fosters intergenerational interactions through shared music-making. Kindermusik Bridges was initially developed and implemented at the Elizabeth and Tab Williams Adult Day Center in Winston-Salem, NC. Anecdotal reports suggest it has positive effects on stress and quality of life in PWD and their caregivers, with caregivers and facilitators observing increased engagement, emotional expression, and stress reduction among PWD. One challenge in assessing effects of Kindermusik Bridges on quality of life and stress is that changes in these factors may be short-lived and difficult or impossible to self-report in a population with dementia. A potential solution to this challenge is ecological momentary assessment (EMA), a set of real-time data collection techniques that reduce recall bias by capturing multiple daily assessments in real time and in the real world (Fanning et al., 2024; Shiffman et al., 2008). However, the use of EMA to collect data from PWD and their caregivers remains limited (van Knippenberg et al., 2017), particularly in community-delivered programs where high levels of researcher oversight are not feasible.

The present study aimed to evaluate the feasibility of using EMA techniques within the context of the Kindermusik Bridges program, and to explore whether EMA could provide insights into the transient and cumulative effects of the program on health-related quality of life in both PWD and their caregivers. To this end, we conducted a 12-week observational study wherein we employed an automated EMA protocol to collect real-time reports on momentary stress and affective valence of PWD and their caregivers as the PWD attended weekly Kindermusik Bridges sessions. To further validate the EMA data, we also collected PWD salivary cortisol samples as a biomarker of stress. We hypothesized that 1) it would be feasible to conduct an EMA protocol and salivary cortisol collection in a community setting, 2) we would be able to detect daily improvements in mood and reduced stress via EMA, but would not see long-term benefits in symptoms on retrospectively administered surveys before and after the 12 weeks of intervention, 3) stress outcomes in PWD would be correlated with stress outcomes in caregivers, and 4) cortisol levels would drop in PWD after Kindermusik Bridges classes.

## 2 Methods

### 2.1 Participants

A total of 14 participants with ADRD and their caregivers consented to participate in this 12-week observational study. Participants were either currently participating in (n = 7) or planning to begin (n = 7) an ongoing Kindermusik Bridges program delivered up to twice weekly within Senior Services’ Elizabeth and Tab Williams Adult Day Center (hereafter “Center”). Eligibility required participants to be over 60, have a dementia diagnosis, attend the Center on program days, and express interest - either personally or through their caregiver - in attending Kindermusik Bridges classes. Participants were also able to attend the Center on days when the program was not being delivered. Caregivers were required to own a smartphone device, avoid travel over the 12-week study period, be willing to provide consent, and be willing to engage in study procedures. Participants received an activity monitor as an incentive for participating but were not otherwise compensated. All caregivers and PWD provided IRB-approved consent or assent prior to participation and collection of any data. All work was conducted with the formal approval of the Wake Forest School of Medicine Institutional Review Board, IRB00050268 and adhered to the principles of the Belmont Report and the Declaration of Helsinki.

### 2.2 Study procedures

Center staff advertised the study to eligible participants and their caregivers. Interested dyads met with study staff to complete written, informed consent and provide demographic information, medical history, medications, and details of the dementia diagnosis. Adverse events were monitored through dyad reports at each assessment visit and by observations from program and Center staff. No adverse events were noted.

#### 2.2.1 EMA procedures

To assess the feasibility of collecting EMA data within the context of the Kindermusik Bridges program, we used a custom automated EMA suite wherein caregivers completed surveys via a smartphone or tablet-compatible web app. Each EMA prompted the caregiver to provide information about the affective valence and stress states of both the caregiver and the PWD. To balance caregiver burden while aiming to explore any beneficial transient or cumulative effects of the program, we aimed for six daily EMAs over 1 week at the study’s start and again after 12 weeks of Kindermusik Bridges participation. On days when participants did not attend the Center, caregivers submitted surveys upon waking (unprompted) and in response to automated text prompts that were sent at random within 2-hour blocks throughout the day. On days when participants attended the Center, surveys were completed at similar time points outside Center hours. While EMA data collection by staff during Center visits was planned, a form error prevented its implementation.

#### 2.2.2 Cortisol procedures

Salivary cortisol data were collected to assess the feasibility of this assessment within the Kindermusik Bridges program, and to examine correlations between this biomarker of stress and caregiver-reported stress and affect. Center staff collected salivary swabs before and after Kindermusik Bridges classes at two time points (Week 1 and Week 12) from ten participants. Samples were obtained using the SalivaBio Children’s Swab (Salimetrics, State College, PA), a synthetic swab designed to improve volume collection and participant compliance and validated for use with salivary cortisol. To ensure accuracy while minimizing participant stress, trained nursing staff familiar to participants collected the samples, with a practice sample taken during Week 0 for acclimation. Research staff froze samples upon collection, which were later batch-shipped on dry ice to Salimetrics for processing. Samples were thawed, vortexed, and centrifuged for 15 min at approximately 3,000 RPM (1,500 x g) before testing via high-sensitivity enzyme immunoassay (Cat. No. 1–3,002). This process required 25 μL of saliva per determination. The assay had a sensitivity of 0.007 μg/dL, a standard curve range from 0.012–3.0 μg/dL, and intra- and inter-assay variation coefficients of 4.60% and 6.00% respectively-meeting Salimetric’s accuracy criteria and NIH reproducibility guidelines.

### 2.3 Description of the intervention

Kindermusik Bridges classes were held at the Center once per week for a duration of 60 min. Traditional Kindermusik classes engage children and parents in music-making activities like clapping, instrument playing, singing, and movement. These sessions blend structured activities, such as playing egg shakers and bells and participating in follow-the-leader movements, with the exploratory experience of listening and relaxing to music. In Kindermusik Bridges classes, older adults are part of the group and actively participate in the activities while interacting with the children and parents. These classes were designed to encourage the older adult to act as a teacher and role model for the children, while the children follow their lead and act as helpers by handing out instruments and props. These classes aim to provide meaningful engagement for older adults through key modifications: 1) Providing older adults with the same instruments and manipulatives as children; 2) Encouraging older adults to model participation, similar to a child’s caregiver in traditional classes; 3) Facilitating structured social interactions, such as children distributing instruments; and 4) Allowing natural moments of shared joy, movement, and connection. While primarily serving PWD, Kinermusik Bridges has also been implemented in settings with participants who have no cognitive impairments, promoting broader intergenerational connections. The music used in Kindermusik Bridges is meant to engage both children and older adults, as it is recorded within a vocal range that supports children’s language development while also featuring familiar traditional Americana tunes that older adults recognize and enjoy (Kindermusik, 2023).

### 2.4 Data collection during intervention

Retrospective questionnaires that are commonly used in dementia research were used to assess caregiver burden, neuropsychiatric symptoms in the PWD, and quality of life for the PWD. Caregiver burden was assessed using the 22-item Zarit Caregiver Burden Scale (Zarit et al., 1980). Behavioral and psychological symptoms of dementia were assessed using the Apathy Evaluation Scale (AES) and the Neuropsychiatric Inventory (NPI). The AES is a brief questionnaire that queries symptoms of apathy (Lueken et al., 2007). The NPI is a structured interview designed to assess neuropsychiatric symptoms in the person with dementia by care partner report (Cummings, 1997). The NPI includes questions about 12 domains of symptoms: delusions, hallucinations, agitation/aggression, depression, anxiety, elation/euphoria, apathy, disinhibition, irritability, aberrant motor activity, sleep, and eating. Scoring for the NPI includes caregiver ratings of each symptom’s severity and frequency that are combined into a total frequency x severity score, and a rating of caregiver distress in response to the symptom. Here we used the frequency x severity score. Quality of life was assessed using the QOL-AD, a brief questionnaire validated for administration to both caregivers and the PWD (Bárrios et al., 2013; Logsdon et al., 2002; Logsdon et al., 2007). We used caregiver scores.

In Week 1 and Week 12, Center staff members recorded daily whether participants engaged in Kindermusik Bridges that day and rated PWD engagement during the class on a 0–4 scale (0 = Highly Engaged, 4 = Not Engaged). A dichotomous variable representing participation and engagement was computed, coding days as one if the participant attended the class and had high engagement (e.g., had a scores of 0-Highly Engaged or 1-Engaged), and 0 if they did not attend or had low engagement (e.g., scores of 2, 3, or 4-Not Engaged).

Caregiver-reported affective valence was assessed via EMA using a modified Feelings Scale (Hardy & Rejeski, 1989), where caregivers rated both their own feelings (“How good or bad do you feel?”) and the PWD’s feelings (“How good or bad does your loved one feel?”) on an 11-point Likert scale (0 = “very bad”, 10 = “very good”). Stress was similarly measured with a single EMA item for caregiver stress (“What is your current level of stress?”) and PWD stress (“What is your loved one’s current level of stress?”) on an 11-point Likert scale (0 = “not at all stressed”, 10 = “very stressed”). That is, caregivers completed four questions on each EMA instance, two about themselves and two about the PWD.

### 2.5 Analyses

Descriptive statistics were used to summarize demographic data and quantify survey completion rates. Given the small sample size, differences between Week 1 and Week 12 questionnaires and cortisol data were compared nonparametrically using Wilcoxan signed rank tests and all data were included. As a preliminary investigation of relationships between momentary and cumulative stress and affect, we fit multilevel linear regression models while accounting for nesting within the individual. Each model first included a random intercept, followed by a series of fixed main effects: time of day (i.e., EMA prompt number), day of the week (0 = Sunday), study week, participant age, time since diagnosis, and Kindermusik Bridges participation on that day.

To test whether we were able to detect daily changes in mood or stress in response to Kindermusik Bridges, separate models were computed for predictions in PWD and caregivers. First, we assessed whether caregiver-reported PWD stress/affect ratings predicted caregiver stress/affect. Models testing whether caregivers improved stress or mood on days when Kindermusik Bridges occurred tested for interactions between time of day, the PWD’s stress/affective state, and whether or not the PWD had participated in Kindermusik Bridges that day. Models testing whether caregiver-reported PWD stress and affect improved on Kindermusik Bridges days included caregiver stress/affect to account for rating bias and tested for interactions between time of day, and participation in Kindermusik Bridges on that day. To assess if we could detect changes between Week one and Week 12 using EMA, we tested an additional model where timepoint (Week 1 and Week 12) was added to the above models. Interactions with *p* values ≤ 0.10 were retained in the model, and statistical significance was established at *p* < 0.05. Significant interactions were visualized by using a median split on continuous predictors when plotting predicted stress or affect values. For each model, we verified the normality of residuals by visually inspecting the residual histogram. To establish linearity, scatterplots were created for each continuous predictor against each dependent variable. Additionally, we used scatterplots of the model’s predicted values against the residuals to establish homoscedasticity. Finally, to investigate whether PWD cortisol was associated with caregiver stress and/or caregiver ratings of PWD stress, we computed Spearman’s rank order correlations between PWD cortisol levels (collected from nine participants) and both average caregiver stress and PWD stress as reported by the caregiver via EMA. Given the pilot nature of this sample, we anchored our interpretation of correlation coefficients using the following common strength categories: |⍴| = 0–0.2: very weak; |⍴| = 0.2–0.4: weak; |⍴| = 0.4–0.6: moderate; |⍴| = 0.6–0.8: strong; |⍴| = 0.8–1: very strong. All EMA analyses were completed in SPSS v 25 (IBM Corp, Armonk, NY, United States) and non-EMA analyses were completed using SAS version 9.4 implemented in SAS Enterprise Guide version 7.19 (SAS Institute, Cary, NC, United States). As this was a feasibility study, no power analysis was conducted and analyzed results should be interpreted as preliminary and requiring replication in a full study.

## 3 Results

### 3.1 Participant characteristics

PWD and caregiver characteristics are displayed in Table 1. The 14 PWD had a mean age of 79.43 ± 9.39 years, 71.4% (10/14) were female, and 78.6% (11/14) were white. Most PWD participants (71.4% (10/14)) were diagnosed with dementia within the past 5 years. Caregivers had a mean age of 52.21, 92.9% (13/14) were female, and 71.4% (10/14) were white. To minimize professional caregiver burden, attendance was only recorded on weeks of data collection. Two Kindermusik classes were offered each week and attendance partly varied based on whether or not participants usually attended the Center on those days. In Week 1, 9 of 14 PWD attended Kindermusik on Day 1 and of the 9 who attended, 7 had engagement scores of 0 or 1 (highly engaged) and 2 had engagement scores of 3 (low engagement). Week 1/Day 2, 6 participants attended, with 5 having engagement scores of 0 or 1 (highly engaged) and 1 having a score of 3 (low engagement). In Week 12, 13 PWD attended Kindermusik on Day 1. Of these 13, 8 had high engagement (scores of 0–1) and 5 had low engagement (scores of 2–3). For Week 12/Day 2, 7 of 14 participants attended, and of these, all 7 were reported to have high engagement.

**TABLE 1:** Participant characteristics.

	Participants (N = 14)	Caregivers (N = 14)
Age, mean (SD)	79.43 (9.39)	52.21 (12.64)
Sex, N (%)		
Female	10 (71.4)	13 (92.9)
Male	4 (28.6)	1 (7.1)
Race, N(%)		
White	11 (78.6)	10 (71.4)
Black	3 (21.4)	3 (21.4)
More than one race	0 (0)	1 (7.1)
Diagnosis, N (%)		
Alzheimer’s disease	5 (35.7)	
Vascular dementia	4 (28.6)	
Other/Unknown	5 (35.7)	
Time since diagnosis, N (%)		
Within 5 years	10 (71.4)	
More than 5 years	3 (21.4)	
Missing	1 (7.1)	
CDR sum of boxes, mean (SD)^a^	1.6 (0.67)	
MMSE, mean (SD)^a^	13.9 (5.0)	

Notes: SD, standard deviation.

^a^ Collected on 12/14 participants.

Example comments for participants with high engagement included “Actively engaged with verbal interaction and movement, encouraged laughter with peers and children” and “Participant came to Kindermusik on her own when she saw children had arrived. Over the course of the 12 weeks, she has been active physically and interacted with a positive attitude with the children. She enjoys the intergenerational atmosphere of the toddlers and adults as demonstrated by her positive attitude, physical interaction and talking to the children. She is receptive to the children’s touch, hugs and Kindermusik activities”. Example comments for participants with low engagement included “Pt. usually does not participate. She will sit and smile as her head turns towards the action in the room. She watches and observes. Normally a reserved person and does n't participate in large groups. However, she spoke to her peers afterwards in a conversation” and “Neutral with minimal hand movements. Mostly observed. Eye-tracking (following the children), smiling.”

### 3.2 Effects of Kindermusik on non-EMA outcomes

In Weeks 1 and 12, caregivers completed questionnaires of outcomes that are commonly measured in studies of caregivers and PWD, including the Zarit Caregiver Burden Scale, AES, NPI, and QOL-AD (Table 2). Overall, there was a significant change in caregiver burden, with an average increase in burden of 7.46 points over the course of the study (std dev = 5.12, *p* < 0.01). No significant changes were observed in apathy, quality of life, or frequency/severity of reported symptoms on the NPI.

**TABLE 2:** Unadjusted mean scores for caregiver questionnaires.

	Week 1		Week 12		
	Mean	Std dev	Mean	Std dev	P-value
Zarit Caregiver Burden Scale	28.64	16.37	34.62	15.84	<0.01*
Apathy Evaluation Scale (AES)	28.33	7.56	28.08	7.33	0.97
Neuropsychiatric Inventory (NPI)	14.43	9.36	22.54	15.52	0.19
QOL-AD	28.69	4.52	27.92	5.09	0.83

Notes: NPI, score reported is frequency x severity; QOL-AD, Quality of Life–Alzheimer’s Disease.

### 3.3 EMA response rates

EMA response rates were examined to assess feasibility of conducting a low-touch EMA protocol in a community setting with caregivers of PWD. There were 1,176 possible EMA responses (14 caregivers completing six daily surveys over two week-long time points), and we received 443 (37.7%) completed surveys. Completion rates were lowest for the first unprompted survey of the day (27%), for lunch-time surveys (30.3%), and for afternoon surveys (25.1%). Other completion rates were 49.2% for late morning surveys, 48.2% for dinnertime surveys, and 47.7% for evening surveys. The very low survey completion rates at lunchtime and afternoon include the planned survey collection by staff that were inadvertently omitted. After excluding the lunchtime and afternoon surveys, the completion rate was 42.9%. Restricting the completion rates to only prompted surveys by excluding the morning survey, the completion rate was 48.4%.

### 3.4 EMA outcomes for caregivers

**FIGURE 1:**
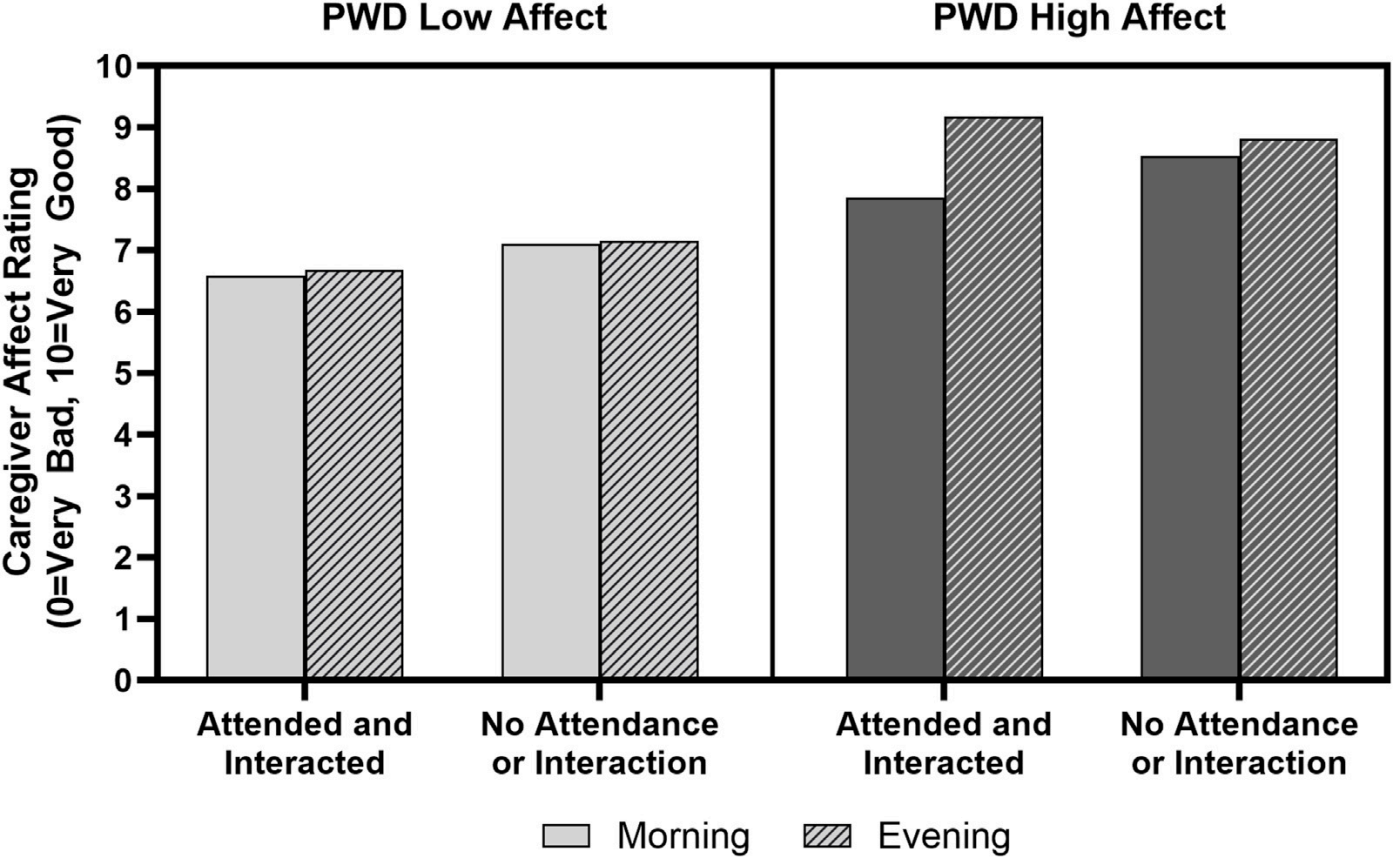
Caregiver affect is a function of the PWD’s affect, time of day, and participation in Kindermusik Bridges. Bars show caregiver affect where higher values indicate more positively valanced affect. Solid bars show affect ratings from the morning and striped bars show affect ratings in the evening. The left box shows caregiver affect in morning and evening when the PWD was rated as having low affect. Regardless of whether the PWD attended and interacted, if the PWD began the day with low affect, the caregiver experienced lower affect throughout the day. In contrast, days when the PWD began the day with high affect, the caregivers’ affect improved between morning and evening if the PWD attended and interacted in Kindermusik. Error bars are too small to display.

Final multilevel models are presented in Table 3. The model of caregiver affect testing the potential daily effect of Kindermusik Bridges on caregiver mood revealed a significant interaction between the PWD’s affect, time of day, and whether the PWD had attended Kindermusik Bridges on that day (B = 0.16, *p* = 0.01). Investigation of a plot of predicted values (Figure 1) indicates that in general, caregiver affect remained steady on days without Kindermusik Bridges participation but tended to increase across the day on days with Kindermusik Bridges participation, particularly when the PWD exhibited higher affect. A significant main effect for time point (B = 0.54, *p* < 0.01) indicated that caregiver affect tended to improve over the 12-week study. There was also a significant main effect for caregiver affect (B = 0.45, *p* < 0.01), such that caregiver ratings of their personal affect were positively correlated with their ratings of the PWD’s affect. Model residuals were negatively skewed, but after this was remedied by applying a reflected inverse transformation to the caregiver affect ratings, this did not affect significance within the model.

**TABLE 3:** Multilevel models for caregiver affect and stress.

Effect in multilevel model	B	*p*
Overall Caregiver Affect		
Time Point	0.54	<0.01*
Day in Week	0.05	0.15
Age	−0.02	0.66
Attended Kindermusik	1.89	0.12
Time Since Diagnosis	−1.09	0.18
PWD’s Affect	0.45	<0.01*
Daily Prompt Number	0.09	0.63
PWD Attendance * PWD’s Affect	−0.31	0.05
PWD Attendance * Daily Prompt Number	−1.15	0.01*
PWD’s Affect * Daily Prompt Number	−0.01	0.79
PWD’s Affect * Daily Prompt Number * PWD Attendance	0.16	0.01*
Overall Caregiver Stress		
Time Point	−0.38	0.04*
Day in Week	−0.04	0.37
Age	0.06	0.17
Attended Kindermusik	0.28	0.41
Time Since Diagnosis	2.48	0.01*
PWD’s Stress	0.46	<0.01*
Daily Prompt Number	−0.09	0.08
PWD Attendance * PWD’s Stress	0.25	0.05
Overall PWD Affect		
Time Point	−0.95	<0.01*
Day in Week	0.04	0.30
Age	0.03	0.42
Attended Kindermusik	−0.66	0.06^+^
Time Since Diagnosis	−0.17	0.81
Daily Prompt Number	0.00	0.93
Caregiver Affect	0.54	<0.01*
Time Point * Attended Kindermusik	1.46	<0.01*
Overall PWD Stress		
Time Point	0.04	0.82
Day in Week	0.00	1.00
Age	−0.02	0.47
Attended Kindermusik	0.04	0.87
Time Since Diagnosis	−0.54	0.35
Daily Prompt Number	0.00	0.96
Caregiver Stress	0.36	<0.01*

The model of caregiver stress also revealed a significant main effect for time point (B = −0.38, *p* = 0.04), such that caregivers tended to report lower stress over the 12-week study. There was also a significant main effect for time since the PWD had received their dementia diagnosis (B = 2.48, *p* = 0.02), such that caregivers of individuals with a longer duration of dementia tended to report higher stress. Additionally, a significant main effect for the caregiver’s perception of the PWD’s stress (B = 0.45, *p* < 0.01) revealed that caregivers felt more stress when they perceived the PWD as having more stress. Model residuals were positively skewed, and after this was remedied by applying an inverse transformation, the transformed model also revealed a significant main effect for time of day (with caregiver stress tending to decrease across the day (*p* < 0.01). A strong positive correlation between average caregiver stress and PWD cortisol was observed at both Week 1 (Spearman’s rho = 0.68) and Week 12 (Spearman’s rho = 0.69).

### 3.5 EMA outcomes for people with dementia

**FIGURE 2:**
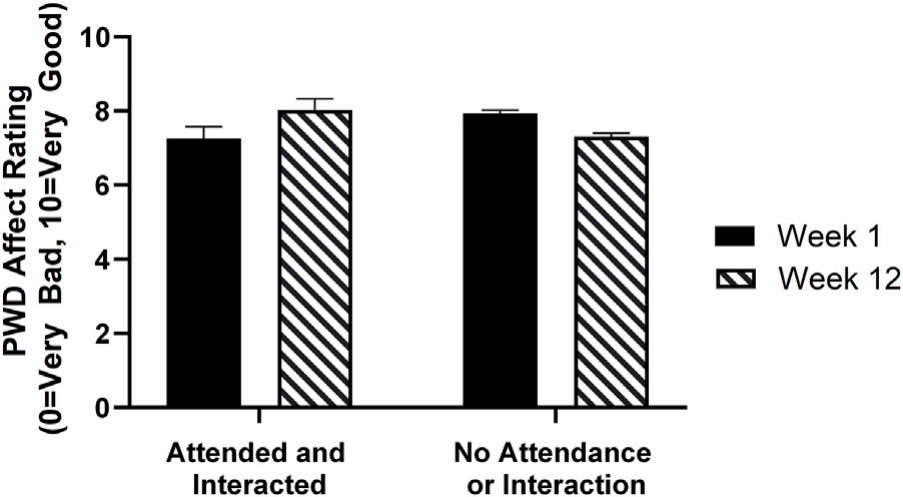
Average predicted caregiver ratings of PWD affect at Week 1 and Week 12 Bars on the left show PWD affect on days when they attended and engaged in Kindermusik Bridges and bars on the right show affect on days they did not attend or interact. Black bars show Week 1 and striped bars show Week 12. At Week 1, caregivers tended to report PWD as having slightly higher affect on days when they did not attend or engage in Kindermusik than on Kindermusik days. However, at the 12-week timepoint, caregivers tended to report the PWD as having more positive affect on days when they participated in Kindermusik Bridges than days when they did not.

In the model to test the effects of Kindermusik Bridges participation on PWD affect, caregiver-reported PWD affect showed a significant interaction between time point and participation in Kindermusik Bridges (B = 1.46, *p* < 0.01), illustrated by Figure 2. At baseline, caregivers tended to report more positively valanced PWD affect on non-Kindermusik days than Kindermusik days, but at Week 12, caregivers tended to report more positively valanced PWD affect on days when they participated in Kindermusik Bridges. There was also a main effect for caregiver affect (B = 0.54, *p* < 0.01), indicating caregiver affect was higher when they perceived the PWD as having higher affect.

As in the caregiver models above, the model of PWD stress revealed a significant main effect for caregiver stress (B = 0.36, *p* < 0.01) such that caregiver ratings of their personal stress correlated positively with their ratings of the PWD’s stress, with no other significant effects. Caregiver ratings of PWD stress displayed a strong positive skew, but after applying a natural log transform as well as a natural log transform while trimming outliers (n = 10), only a mild positive skew remained (skewness Z = 3.78). Only PWD stress was related to caregiver stress across each model (*p* < 0.01).

### 3.6 Cortisol outcomes for people with dementia

Salivary cortisol was sampled immediately before and after Kindermusik Bridges classes in Weeks 1 and 12 (Supplementary Figure S1) in a subset of participants. In Week 1, 6 of 9 participants from whom cortisol was collected showed lower cortisol levels after attending class, and in Week 12, 6 of 10 participants showed lower cortisol levels after attending class. However, there were no statistically significant changes in cortisol levels between the beginning and end of class for either week (Week 1: Mean change = −0.003 μg/dL, std dev = 0.05, *p* = 0.51; Week 12: Mean change = −0.003 μg/dL, std dev = 0.07, *p* = 0.82). Cortisol levels also did not differ significantly between Week 1 and Week 12 for pre-class (Mean change = 0.02 μg/dL, std dev = 0.07, *p* = 0.36) or post-class (Mean change = 0.02 μg/dL, std dev = 0.06, *p* = 0.36). Additionally, when relating cortisol levels to caregiver-reported stress of PWD, there was a moderate positive correlation between PWD cortisol and average caregiver-reported PWD stress at Week 1 (Spearman’s rho = 0.57; *p* = 0.11) but not at Week 12.

## 4 Discussion

This observational pilot feasibility study provided a few key outcomes to inform similar future research. The first aim of the study was to determine the feasibility of integrating EMAs collected by caregivers and staff at a community Center into the Kindermusik Bridges program. Despite accounting for data lost due to a protocol error, we observed low survey completion rates (∼50%) even on prompted EMA surveys. Other EMA studies of caregivers with low researcher oversight have reported comparable rates; for example, a recent study observing cancer caregivers who completed 8 daily assessments for a week had a 59% completion rate (Shaffer et al., 2021). Notably, caregivers of PWD often report higher burden and lower quality of life compared to cancer caregivers (Moraes and Silva, 2009). One potential strategy to enhance response rates for surveys collected from burdened caregivers is to make contact early in the measurement window when additional staff support is available. In an EMA study of mood among spousal caregivers of PWD that involved 10 daily surveys for 6 days, van Knippenberg and colleagues (2017) placed telephone calls to each caregiver on the second assessment day to query about any study difficulties, and a second call was placed later in the week for additional compliance support as needed. The researchers reported an average completion rate of 78.8%. Others have suggested minimizing prompt frequency (e.g., via a single daily survey) to minimize burden (Potts et al., 2020). Further research is needed to explore if low-touch support methods like automated phone or in-person reminders can improve EMA completion rates without adding significant staff costs. Indeed, an emerging area of experimental research involves leveraging factorial research designs to examine factors associated with enhanced EMA completion rates (Businelle et al., 2024), though to date none have been conducted with caregivers of PWD. Future research is warranted to focus on understanding (1) how many daily surveys are required to understand relationships between participation in a class like Kindermusik Bridges and psychological states like stress and affect; (2) how the rate and pattern of missingness impact these questions; and (3) how to enhance completion rates among this highly burdened population.

Second, we hypothesized that it would be possible to detect daily improvements in affect and stress using EMA, but not changes in symptoms using routinely administered questionnaires. Caution is needed when interpreting results of the models predicting PWD and caregiver affect and stress, given the small sample size, pilot study design, and low survey completion rates. Acknowledging these limitations, EMA provided novel preliminary insights into the daily affective and stress states of PWD attending a Kindermusik Bridges program and their caregivers. We observed that caregiver affect slightly improved on days when their PWD interacted in the Kindermusik Bridges class, especially when the caregivers perceived the PWD as having higher affective valence. Additionally, caregiver affect tended to be higher on Kindermusik days relative to non-Kindermusik days over time, suggesting a potentially cumulative effect of PWD Kindermusik attendance in caregivers. These results are especially important for those interested in sustaining caregiver quality of life, as affect plays an important role in perceptions of quality of life (van Leeuwen et al., 2012). In contrast to EMA results, commonly used questionnaires tracking caregiver stress, PWD symptoms, and quality of life showed little change, except an increase in caregiver burden. Caregiver burden can include factors outside the sphere of influence of the Center or the intervention, such as financial strain or caregiver health, and is expected to increase over time. The duration of effects highlights the value of momentary assessments relative to traditional retrospective recall instruments for assessing affective outcomes in PWD. Overall, these findings supported the idea that EMA data collection might be more sensitive to the effects of intervention in a population with poor ability to self-report and a condition expected to worsen over time.

As noted in the introduction, improving quality of life is a major focus of care in ADRD for both the caregivers and the PWD. However, as dementia is a progressive disease that results in impairment of the ability to self-report on affective states, assessing whether interventions result in more positive mood states for PWD is challenging. Here, we observed that caregiver reports of the PWD’s affective states improved only on the day of the Kindermusik Bridges program. That is, effects were not sustained across multiple days. This effect may be due to the low dose of the intervention (one class per week for 60 min). It is also possible that akin to a medication that must be taken daily, effects are transient and confined to the day of participation-again suggesting that additional benefits may come from more frequent participation and that additional work investigating the impact of more frequent classes is warranted. Finally, it is important to note that positive effects were associated with more active participation in the Kindermusik Bridges class. Future studies of Kindermusik Bridges may investigate whether actively working to engage individuals in each class enhances the impact of the program. Additionally, replicating this research in nursing homes or assisted living facilities could help determine whether the structured environment and additional staff support in these settings may influence participant engagement and intervention effectiveness, while also contributing to the growing literature on implementing music-based interventions for PWD into residential long-term care (Amano et al., 2022; Tompkins et al., 2020).

Third, we hypothesized that stress outcomes would be correlated between caregivers and PWD. Our preliminary analyses provide support for the intuitive notion that the day-to-day psychological state of the caregiver and PWD are closely linked, as perceptions of the PWD’s affect and stress were strong predictors of caregiver affect and stress. In addition, baseline salivary cortisol collected from the PWD (González-Cabrera et al., 2014) was correlated with PWD stress reported by the caregiver. Lastly, we hypothesized that cortisol levels would drop immediately after Kindermusik Bridges class in PWD due to the positive interactions of the class. Our data did not support this hypothesis.

### 4.1 Additional limitations

In addition to limitations described above, this convenience sample was predominantly White and female, limiting the generalizability of findings to caregiving relationships under different socioeconomic contexts. As some participants were already enrolled in Kindermusik Bridges, long-term changes may be underestimated. Future studies would benefit from recruiting a representative sample with no previous experience with the program, including a control or referent condition, and using a larger sample size. Lastly, EMA data collection during Center visits was inadvertently missed due to a form error, so future research should involve more on-site study staff to ensure continuous data collection.

### 4.2 Future directions

Overall, this study offers valuable insights into using automated EMA with caregivers of PWD, and the resulting data provides rationale for studying dynamic psychological outcomes between caregivers and their care partners. Additional preparatory work is warranted to identify best practices for enhancing completion rates of EMA among caregivers of PWD in the context of a community-delivered intergenerational program.

## 5 Conclusion

Dementia is a complex chronic condition that affects the daily health-related quality of life of both the person living with the disease and their caregivers. EMA offers the opportunity to investigate the highly dynamic nature of factors that affect health-related quality of life, such as stress and affect, and this may be especially important for capturing positive but short-lived benefits in response to promising community movement and music programs. Additional research is required to determine how to best support EMA survey completion among burdened caregivers, and to investigate the impact on health-related quality of life of more frequent involvement in Kindermusik Bridges.

## Data Availability

The raw data supporting the conclusions of this article will be made available by the authors, without undue reservation.
