# Supplementary material for: Effects of an intergenerational Kindermusik class on stress and affect in older adults with dementia and their caregivers: a pilot study using ecological momentary assessment
Source: Front Aging. 2025 Jun 17;6:1448293. doi: 10.3389/fragi.2025.1448293 (PMC12209361; doi:10.3389/fragi.2025.1448293)
Supplement: Supplementary file 1 [file DataSheet1.docx]

**Supplementary Figure 1. Individual changes in cortisol before and after Kindermusik class in Week 1 and Week 12.**

**
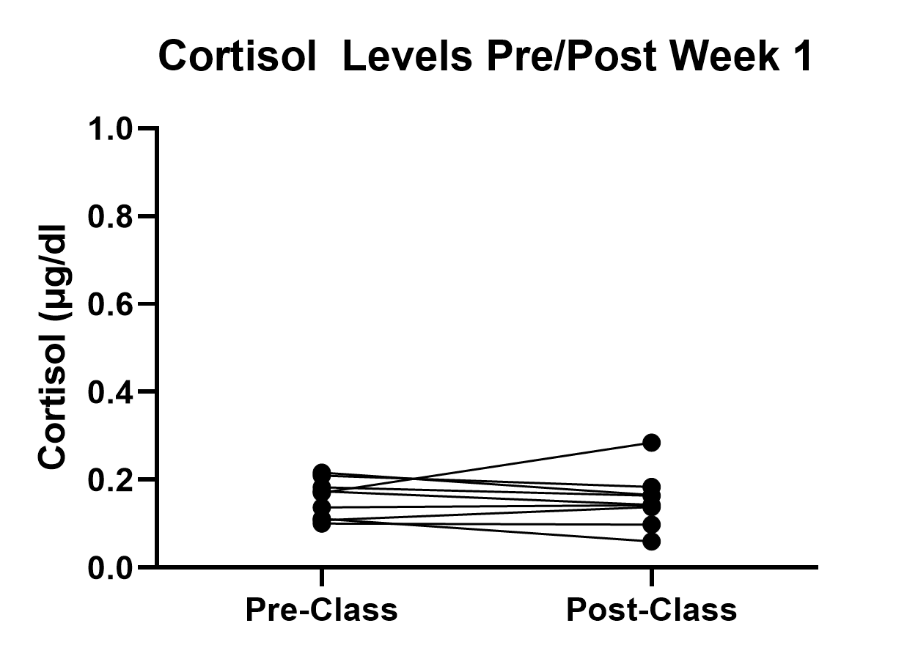

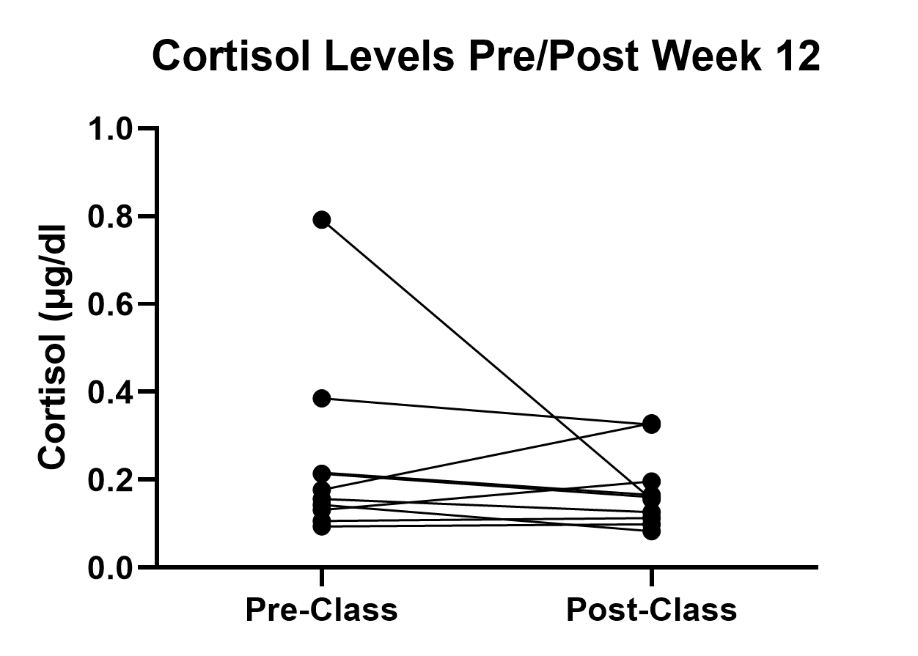
**

**Supplementary Figure 1 Caption.** In Week 1, 6 of 9 participants from whom cortisol was collected showed lower cortisol levels after attending class, and in Week 12, 6 of 10 participants showed lower cortisol levels after attending class. However, there were no statistically significant changes in cortisol levels between the beginning and end of class for either week
